# Engineering the Osmotic State of *Pseudomonas putida* KT2440 for Efficient Cell Disruption and Downstream Processing of Poly(3-Hydroxyalkanoates)

**DOI:** 10.3389/fbioe.2020.00161

**Published:** 2020-03-05

**Authors:** Ignacio Poblete-Castro, Carla Aravena-Carrasco, Matias Orellana-Saez, Nicolás Pacheco, Alex Cabrera, José Manuel Borrero-de Acuña

**Affiliations:** ^1^Biosystems Engineering Laboratory, Center for Bioinformatics and Integrative Biology, Faculty of Life Sciences, Universidad Andres Bello, Santiago, Chile; ^2^Unidad de Citometría de Flujo, Facultad de Ciencias Biológicas, Pontificia Universidad Católica de Chile, Santiago, Chile; ^3^Institute of Microbiology, Technische Universität Braunschweig, Braunschweig, Germany; ^4^Braunschweig Integrated Centre of Systems Biology, Technische Universität Braunschweig, Braunschweig, Germany

**Keywords:** *Pseudomonas putida*, cell lysis, porins, MscL, osmotic stress, poly(3-hydroxyalkanoates), PHA recovery

## Abstract

In the last decade, the development of novel programmable cell lytic systems based on different inducible genetic constructs like the holin–endolysin and lysozyme appears as a promising alternative to circumvent the use of costly enzymes and mechanical disrupters for downstream processing of intracellular microbial products. Despite the advances, upon activation of these systems the cellular disruption of the biocatalyst occurs in an extended period, thus delaying the recovery of poly(3-hydroxyalkanoate) (PHA). Herein the osmotic state of *Pseudomonas putida* KT2440 was engineered by inactivating the inner-membrane residing rescue valve MscL, which is responsible mainly for circumventing low-osmolarity challenges. Then the major outer membrane porin OprF and the specific porin OprE were overproduced during PHA producing conditions on decanoate-grown cells. The engineered *P. putida* strains carrying each porin showed no impairment on growth rate and final biomass and PHA yield after 48 h cultivation. Expression of both porins in tandem in the mutant strain KTΔ*mscL*-*oprFE* led to a slight reduction of the biomass synthesis (∼10%) but higher PHA accumulation (%wt) relative to the cell dry mass. Each strain was then challenged to an osmotic upshift for 1 h and subsequently to a rapid passage to a hypotonic condition where the membrane stability of the KTΔ*mscL*-*oprFE* suffered damage, resulting in a rapid reduction of cell viability. Cell disruption accounted for >95% of the cell population within 3 h as reported by colony forming units (CFU), FACS analyses, and transmission electron microscopy. PHA recovery yielded 94.2% of the biosynthesized biopolymer displaying no significant alterations on the final monomer composition. This study can serve as an efficient genetic platform for the recovery of any microbial intracellular compound allowing less unit operation steps for cellular disruption.

## Introduction

Bio-based plastics represent an attractive solution to circumvent the environmental burden posed by conventional plastics derived from fossil resources (Poblete-Castro et al., 2013). Many species of bacteria can synthesize a myriad of intracellular polymer forms ranging from polyphosphates (PolyP) (Varas et al., 2017) to sugar-containing monomers (Moradali et al., 2015). One of the most studied biopolymers in microbes is the family of poly(3-hydroxyalkanoate) (PHA), carbon inclusion bodies displaying similar physical properties to petroleum-based plastics such as polypropylene (PP). PHA diverge in the length of the monomer carbon chain comprising various polyesters such as short-chain-length (*scl*-PHA) and medium-chain-length PHA (*mcl*-PHA) (Volova et al., 2017). The PHA synthase (PhaC) catalyzes the polymerization process enabling the incorporation of different monomers in the polymeric chain of the polyoxoester, which finally results in distinct thermophysical attributes, thus expanding the number of products and application (Volova et al., 2017; Pacheco et al., 2019). *Pseudomonas putida* KT2440 is an archetype bacterium for the production of mcl-PHA (Poblete-Castro et al., 2017; Mozejko-Ciesielska et al., 2019), where their accumulation in the cell usually occurs under carbon excess accompanied by the limitation of inorganic compounds such as N, O_2_, or P (Prieto et al., 2016).

The highly versatile metabolism *of P. putida* KT2440 endows the bacterium to harness a wide spectrum of substrates – waste oils, raw glycerol, lignin derivatives and further aromatics – which allows reducing the production costs (Poblete-Castro et al., 2014; Kohlstedt et al., 2018; Borrero-De Acuña et al., 2019; Poblete-Castro et al., 2019). Additionally, downstream processing procedures also contribute to increasing the overall production expenses due to the use of lytic enzymes, mechanical cell disruptors, high pressure, or temperature for recovery of the added-value products from the intracellular space of the cell. In the last decade, researchers have developed several programmable genetic systems to induce cell disruption upon command relying on holin–endolysin binary action (Liu and Curtiss, 2009; Martinez et al., 2011; Tamekou Lacmata et al., 2017) or lysozyme translocation into the periplasmic space to enable degradation of the peptidoglycan layer (Borrero-De Acuna et al., 2017).

These inducible tools find applications not only in the biopolymer manufacturer sector but also for intracellular protein release and fatty acid extraction. Despite the advances in autolysis cell technology, there are however still some pitfalls: (i) low titers of the final biopolymer, (ii) biopolymer-producing conditions harm the expression of the genetic circuits, and (iii) the cell disruption process is very slow (Borrero-De Acuna et al., 2017). With the aim of bypassing these drawbacks the construction of a programmable cell lysis system relying on the drastic shift of the osmotic state of *P. putida* cells was sought. It was hypothesized that rapid osmotic alteration of the cell milieu from hypertonic to hypotonic conditions and further depriving *P. putida* of specific response mechanisms designed to counteract osmotic stress would lead to extensive cell disruption in a shorter time.

In Gram-negative bacteria, different molecular elements localized to the inner and outer membranes maintain the cell turgor. Among them, porin proteins are water-filled channels residing in the outer membranes of Gram-negative bacteria that preserve the osmotic equilibrium in ever-changing environments as well as translocating a vast array of solutes (Hancock and Brinkman, 2002). So far, four categories of porins are known: non-specific porins, substrate-specific porins, gated porins (translocate ligands in an energy-dependent fashion), and efflux porins (Hancock and Brinkman, 2002). In pseudomonads, the major non-specific outer membrane porin is the OprF, which encloses a large N-terminal beta-barrel channel allowing the uptake of a broad range of solutes and a periplasmic C-terminal domain rich in alpha-helices (Sugawara et al., 2006). This pleiotropic trans-outer-membrane porin is to a large extent responsible for cell wall permeability and integrity and further contributes to the maintenance of the cell shape. Besides ion incorporation (weakly biased to cations), it is also responsible for the passage of low molecular mass sugars, toluene, and nitrate/nitrite (Chevalier et al., 2017).

Conversely to OprF, anaerobic condition induces the expression of the narrow channel and specific substrate-binding porin OprE (Jaouen et al., 2006). Although showing poor conductivity, the ionophore properties of OprE in *P. fluorescens* point toward a specific cation selectivity and allow diffusion of small ions (Obara and Nakae, 1992; Jaouen et al., 2006). As *P. putida* inefficiently thrives under oxygen-depleted environments the body of knowledge on how gene expression of the *oprE* remains still elusive. Moderate expression of *oprE* has been visualized by Microarray experiments when cells are grown on minimal medium with succinate as a carbon source (Hancock and Brinkman, 2002).

On the other hand, mechanosensitive channels (MscL) have proven essential in both prokaryotes and eukaryotes for regulating the cell turgor (Wang et al., 2014). The ubiquitous MscL of large conductance MscL is a conserved membrane-embedded valve, where mechanical stress cues mediate its response (Sukharev et al., 2001). In bacterial cells they act as safety valves opening their pores upon sudden hypo-osmotic shock to relieve the pressure (Colombo et al., 2003). Gating of the MscL is controlled by high external pressure (∼10 mN/m) that prompts the non-selective pore to open permitting permeabilization to various ions and small organic osmolytes (Najem et al., 2015). In bacteria, its inner membrane settlement represents the last barrier resort of the cell that prevents them from lysing.

In this study, it was hypothesized that the concomitant overproduction of the porins OprF and OprE in *P. putida* and addition of increasing amounts of salt would impose a severe osmotic stress consequently leading to outer membrane perturbation. It was found that aggravation of the osmotic distress endured by the cells via the drastic hypotonic shift in MscL devoid strains by adding distilled water. Our results show that this combined strategy provoked >95% cell lysis within 3 h as recorded by CFU counting and FACS analysis. Furthermore, the system functioned well under PHA-producing conditions, reaching a recovery of nearly 94% of the synthesized mcl-PHA.

## Results

### Rational Approach and Assembly of the Auto-Inducible Cell Disruption System

**FIGURE 1:**
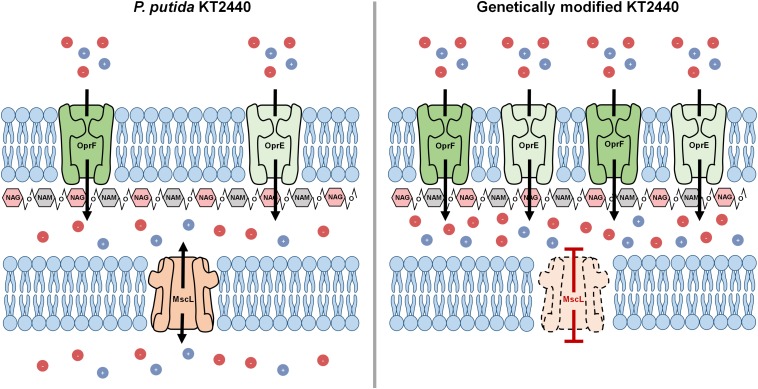
Autolysis model illustrating the binary action of hyper- to hypo-tonic shock in the engineered KT2440 strain.

The MscL is a relief valve that plays a crucial role during hypotonic shock in *Pseudomonas* species. Cell exposure to an external drop of osmolarity immediately triggers MscL channel production, which in consequence permits homeostatic ion release toward the outside counteracting thereby osmotic shock. It was presumed that disruption of the *mscL* gene of *P. putida* (PP_4645) would lead to deterioration of the inner membrane when cells endure hypotonic shock (Figure 1). Therefore, deletion of the ORF encoding for the MscL was addressed by homologous recombination leading to a scarless mutation as specified in the section “Materials and Methods.” The resulting strain is hereafter named KTΔ*mscL.* Cell turgor also relies on an array of outer membrane porins, which incorporate ions into the bacterial periplasm upon demand. Altering the abundance of outer membrane-residing porins was speculated to result in drastic impairments on osmotic stress for *P. putida* and subsequently provoke cell wall damage (Figure 1). To utterly ensure imbalance of the cell wall osmolarity two porins were selected for overproduction. Firstly, the major outer membrane porin (OprF) encoded in the ORF PP_2089 was targeted (Figure 1). The second channel taken into analysis was the outer membrane integral porin OprE (PP_0234), which is normally produced under anoxygenic conditions in other *Pseudomonas* strains (Figure 1). In line with this, the *oprF* and *oprE* genes including Shine-Dalgarno sequences (Table 1) were PCR-amplified from single colonies and introduced separately into the Isopropyl β-d-1-thiogalactopyranoside (IPTG)-inducible vector pSEVA634. Each plasmid was further transferred into both KT2440 and KTΔ*msc*L strain giving rise to the following recombinant strains: KT-*oprF*, KT-*oprE*, KTΔ*mscL*-*oprF*, and KTΔ*mscL-oprE*. Finally, both porin-encoding genes *oprF* and *oprE* were spliced in the above given order and ligated into pSEVA634. Subsequently, this vector (pSEVA634-*oprFE*) was introduced by mating into KT2440 and KTΔ*mscL* originating the KT-*oprFE* and KTΔ*mscL*-*oprFE* strains, respectively.

**TABLE 1:** Strains, vectors, and oligos employed throughout this study.

Strain/vector	Features^*a*^	Source/Reference
*Pseudomonas putida*		
KT2440	Wild-type strain derived from *P. putida* mt-2, devoid of the pWW0 TOL plasmid	(DSMZ)
KTΔ*mscL*	KT2440 deletion mutant lacking the *mscL* gene	This study
KT-*oprF*	KT2440 strain bearing the pSEVA634-*oprF* plasmid	This study
KT-*oprE*	KT2440 strain bearing the pSEVA634-*oprE* plasmid	This study
KT-*oprFE*	KT2440 strain bearing the pSEVA634-*oprFE* plasmid	This study
KTΔ*mscL-oprF*	KT2440ΔmscL mutant strain bearing the pSEVA634-*oprF* plasmid	This study
KTΔ*mscL-oprE*	KT2440ΔmscL mutant strain bearing the pSEVA634-*oprE* plasmid	This study
KTΔ*mscL-oprFE*	KT2440ΔmscL mutant strain bearing the pSEVA634-*oprFE* plasmid	This study
*Escherichia coli*		
DH5α	F–Φ80*lacZ*ΔM15 Δ(*lacZYA-arg*F) U169 *rec*A1 *end*A1 *hsd*R17 (rK–, mK+) *pho*A *sup*E44 λ– *thi*-1 *gyr*A96 *rel*A1	ThermoFisher, Scientific, Darmstadt, Germany
DH5α(λpir	*sup* E44, Δ*lacU169* (Φ*lac*ZΔM15), *recA1*, *endA1*, *hsdR17*, *thi-1*, *gyrA96*, *relA1*, λpir phage lysogen	Biomedal, Seville, Spain
HB101	Helper strain; F– λ– *hsdS20*(rB– mB–) *recA13 leuB6*(Am) *araC14* Δ(gpt- proA)62 *lacY1 galK2*(Oc) *xyl-*5 *mtl*-1 *thiE1 rpsL20* (Sm^R^) *glnX44*(AS)	Benedetti et al., 2016
Plasmids		
pJET1.2	Ap^R^; *oriV* (pMB1) Plasmid employed for subcloning steps	ThermoFisher, Scientific, Darmstadt, Germany
pRK600	Cm^R^; *oriV* (ColE1), tra+ mob+, RK2-based plasmid	Benedetti et al., 2016
pSEVA212	Km^R^; *oriV* (R6K), Sce-I RS. *P. putida* non-replicative vector	Martinez-Garcia et al., 2015
pSEVA628	Gm^R^; *oriV* (RK2), *xylS*-Pm → SceI	Martinez-Garcia et al., 2015
pSEVA634	Gm^R^; *oriV* (RK2), *lacIq*-P*trc*	Martinez-Garcia et al., 2015
pJET1.2-*mscl*UPDW	Ap^R^; pJET1.2 plasmid bearing the fused up- and downstream arms of the *mscL* gene	This study
pSEVA212-*mscl*UPDW	Km^R^; pSEVA212 harboring the spliced up- and downstream flanks of the *mscL* gene	This study
pSEVA634-*oprF*	pSEVA634 plasmid enclosing the major outer membrane porin *oprF* gene	This study
pSEVA634-*oprE*	pSEVA634 plasmid harboring anaerobically induced outer membrane porin *oprE* gene	This study
pSEVA634-oprFE	pSEVA634 plasmid bearing both the *oprF* and *oprE* genes	This study
Oligos (5′ 3′)		
*mscl*UpFw	GAATTCGCAATGTTGCACAAGGTCTG	ThermoFisher, Scientific, Darmstadt, Germany
*mscl*UpRv	ACTGGACTCTTCGCCAGCTTTGGTTCCTTGTAACAAAAGGT	ThermoFisher, Scientific, Darmstadt, Germany
*mscl*DwFw	GCTGGCGAAGAGTCCAGT	ThermoFisher, Scientific, Darmstadt, Germany
*mscl*DwRv	GGATCCGGAGGTCGTGACCTGTGG	ThermoFisher, Scientific, Darmstadt, Germany
*mscl*KOFw	GCATGCTCAACGAGTTCAAG	ThermoFisher, Scientific, Darmstadt, Germany
*mscl*KORv	CGATTCTGGTTCTGCGTCTT	ThermoFisher, Scientific, Darmstadt, Germany
*oprF*Fw	GGATCC*AGGAGG*AAAAACATATGAAACTGAAAAACACCTTGG	ThermoFisher, Scientific, Darmstadt, Germany
*oprF*Rv	TCTAGATTACTTGGCCTGGGCTTCTA	ThermoFisher, Scientific, Darmstadt, Germany
*oprE*Fw	TCTAGA*AGGAGG*AAAAACATATGTACAAGTCCAGCCTGGCTC	ThermoFisher, Scientific, Darmstadt, Germany
*oprE*Rv	AAGCTTTTACAGGAAGTTGTAAGTGTAGT	ThermoFisher, Scientific, Darmstadt, Germany

*^*a*^Abbreviations shown in this table: Cm^*R*^, chloramphenicol resistance cassette; Km^*R*^, kanamycin resistance cassette; Gm^*R*^, gentamycin resistance cassette; Ap^*R*^, ampicillin resistance cassette; I-SceI, SceI endonuclease from *Saccharomyces cerevisiae*; *xylS*-Pm, genetic circuit inducible upon methyl benzoate addition; *lacIq*-P*trc*, genetic system governed by IPTG induction. Restriction sites are underlined. Shine-Dalgarno sequences are shown in italics.*

### Cell Growth and Membrane Assays of the Engineered and Wild-Type Strains

**FIGURE 2:**
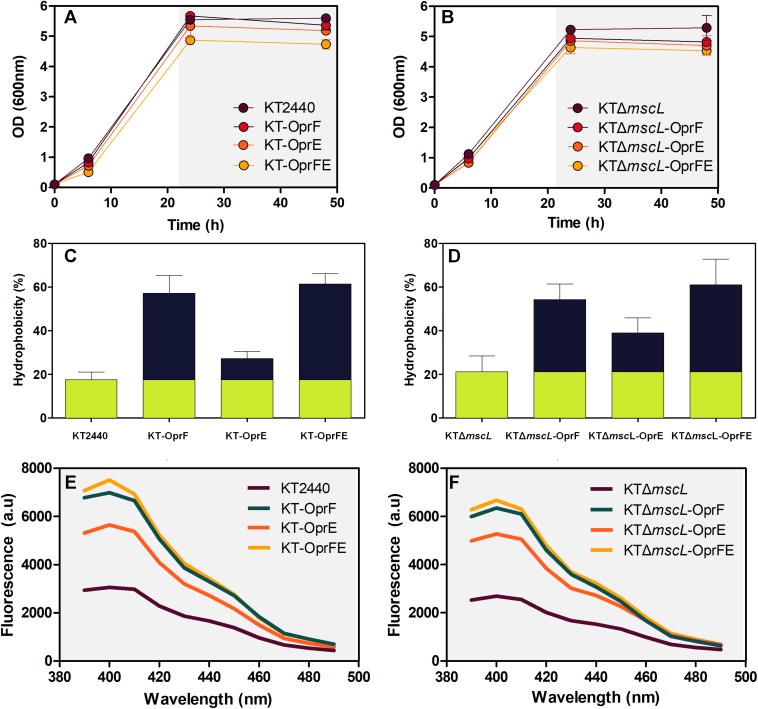
Growth behavior under PHA-producing conditions on decanoate (20 mM) **(A,B)**, overall membrane hydrophobicity **(C,D)**, and permeability **(E,F)** of the engineered *P. putida* strains.

Firstly, it was evaluated whether inactivation of the *mscL* gene and overexpression of the porin genes exerted a detrimental effect on bacterial growth of all constructed strains. Frequently basal transcriptional leakage of plasmidial genes or gene suppression generates a diminished biomass synthesis that will lastly affect PHA accumulation (Guamán et al., 2018). The growth rate of the genetically modified KT2440 strains, namely KT-*oprF*, KT-*oprE*, and KT-*oprFE* did not vary significantly compared with KT2440 wild-type using decanoate (20 mM) as the sole carbon source in minimal salt medium (Figure 2A). Nor deletion of the *mscL* gene appears to influence biomass production negatively (Figures 2A,B), neither did the introduction of the porin genes into this knockout strain, before and after activation of the system via IPTG (1 mM) at 30 h cultivation (Figure 2B). The length of the cultivation was 48 h, as PHA synthesis reached maximal values. At this time point, the hydrophobicity and permeability of the cells were evaluated among strains. The porins locate in the outer membrane of bacteria comprise hydrophobic amino acids and hydrophilic residues. Thus, overexpression of the *oprF* and *oprE* might render the membrane more hydrophobic (Delcour, 2009). Figures 2C,D show that the KT2440 strains carrying the porin proteins boosted the hydrophobicity of the membrane by more than threefold in comparison to the parental and the mutant KTΔ*mscL* strain. The OprF protein contributed the most to this process, as this is the major water-filling porin in Pseudomonas (Chevalier et al., 2017). Consistent with the enhanced hydrophobicity of the cell’s surface of the engineered KT2440 strains, changes in the uptake of *N*-phenyl-1-naphtylamina (NPN) were found, a suitable compound to assess the permeability barrier of the membrane (Helander and Mattila-Sandholm, 2000). This analysis confirms the key role that both porins play in the degree of permeability of the bacterial envelop since the fluorescence intensity is clearly higher for the recombinant strains than that of *P. putida* KT2440 (Figures 2E,F).

### Triggering Cell Disruption in the Engineered *P. putida* KT2440 Strains Using a Coordinated Hypertonic and Hypotonic Treatment

**FIGURE 3:**
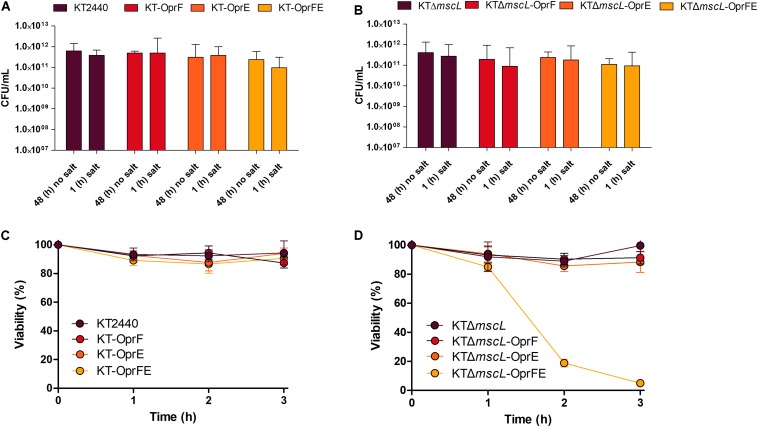
Cell survival expressed as CFU counts per mL after osmotic upshift **(A,B)** and cell viability reflected in percentage (%) over the initial cell density over 3 h **(C,D)** of each genetically modified and parental strain.

Afterward, the osmolarity of cultures was increased by adding NaCl to attain a final concentration of 10 (g/L). After 1 h, CFU exhibited minor cell viability variations in all tested bacterial strains (Figures 3A,B). Later, the entire culture broth was centrifuged, the supernatant discarded, and finally the cell pellet resuspended in the same amount of volume using distilled water, causing thereby a hypoosmotic shock. Strikingly, while the hypotonic pressure did not seem to affect cell survival on most of the strains (Figure 3C), the KTΔ*mscL*-*oprFE* showed substantial cell death (Figure 3D). After imposing hypoosmotic shock cell population diminished by 20, 80, and 96% after 1, 2, and 3 h, respectively (Figure 3D). Thus, the concerted pressure of both OprF and OprE porins and the abolishment of the MscL safe valve are required to achieve the desired impact on cell viability. In contrast the individual action of each porin did not apply sufficient osmotic pressure on the engineered strains to account for enhanced cell mortality rates.

### Assessment of Cellular Disruption by FACS Analysis

**FIGURE 4:**
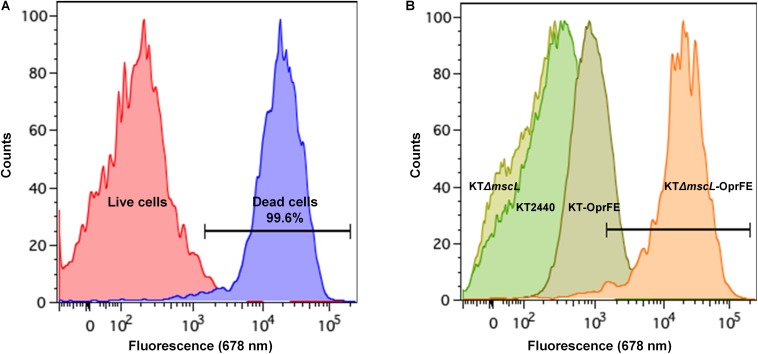
FACS analysis by LIVE/DEATH staining. Cell death and live gates after heat-cold treatment are shown on the left side **(A)** while cell disruption of KT220 wild type, KTΔ*mscL*, KT-*oprFE*, and KTΔ*mscL*-*oprFE* strains is displayed on the right panel **(B)**.

Although cell death in the engineered KTΔ*mscL-oprFE* strain by CFU counts was confirmed, this fact does not directly correlate with cell membrane disruption, which is essential for downstream PHA recovery. Therefore, cell death and membrane integrity were assessed by LIVE/DEATH cell discrimination analysis via FACS employing the non-permeable DNA intercalating agent propidium iodide. This reagent can dye the chromosome solely when the cell membrane is damaged. First, non-lysed cells taken in the middle of the exponential growth phase were recorded in order to establish a threshold for living cells. As depicted in Figure 4A, intact cells present almost no fluorescence. In a separate control experiment to set up a death gate of cells, KT2440 cells underwent rapid shifts of heat-cold shock as described in the section “Materials and Methods.” After five cycles of heat-shock treatment, 99.6% of the cell population was estimated dead spanning the fluorescence range of 5 × 10^3^ to 10^5^ (Figure 4A). Cell viability analysis revealed no apparent changes in membrane permeability of intact cells between KT2440 and the mutant *mscL* strain as they were superimposed (Figure 4B). When the autolytic system was induced in the KT-*oprFE* strain a 21% of cell membrane disturbance was brought about (Figure 4B). Finally, the porin-MscL dual system was triggered in the KTΔ*mscL*-*oprFE* strain resulting in 96.3% cell disruption and death (Figure 3B). This outcome is practically comparable to the total amount of lysed cells after heat-cold shock treatment endurance.

### PHA Yield, Composition, and Recovery

**FIGURE 5:**
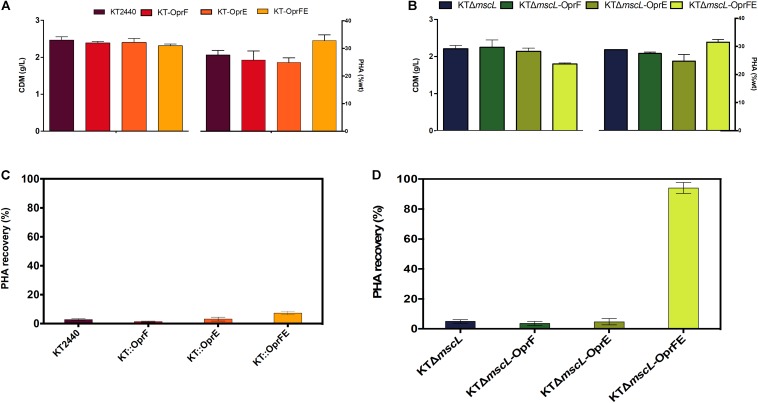
Registration of cell dry mass (CDM) in g/L and PHA accumulation (%wt) of the employed strains **(A,B)** and PHA recovery **(C,D)** of the genetically modified and parental KT2440 strains.

Once the efficiency of the lytic genetic system was verified, the biomass synthesis, PHA yields, and monomer composition was evaluated in the genetically modified *Pseudomonas* strains. The wild-type and the engineered strains displayed nearly equal biomass titers of about 2.5 (g/L) according to their CDM (Figures 5A,B). Hence, it appears that the genome editing and carriage of recombinant porin genes did not impact detrimentally cellular formation. The KTΔ*mscL* deletion mutant produced 2.3 (g/L) and amassed 31.5% of their CDM as PHA (Figures 5A,B), showing similar values to the ones already reported (Oliva-Arancibia et al., 2017). *P. putida* strains harboring the *oprF* or *oprE* constructs trigger a slight decline of about 2 and 11 PHA (%wt), respectively (Figures 5A,B). Interestingly, the KT-*oprFE* and KTΔ*mscL*-*oprFE* strains accumulated almost 10% more PHA (%wt) than their parental strains (Figures 5A,B). By inspecting the monomer composition of the biosynthesized PHA via GC–MS, no considerable variation among the *P. putida* strains was found, being 3-hydroxyhexanoate (C6) the minor monomer and 3-hydroxyoctanoate (C8) and 3-hydroxydecanoate (C10) almost equally distributed in the polymeric chain (Table 2).

**TABLE 2:** Monomer composition of the biopolymers produced by the engineered *P. putida* strains.

Strain	Monomer composition (%)		
	
	C6	C8	C10
KT2440	6.1 ± 0.2	47.8 ± 1.4	46.1 ± 1.9
KT-*oprF*	7.2 ± 0.3	42.8 ± 1.6	50.0 ± 1.1
KT-*oprE*	4.2 ± 0.1	52.6 ± 0.9	43.2 ± 0.5
KT-*oprFE*	4.8 ± 0.6	46.7 ± 1.7	48.5 ± 1.2
KTΔ*mscL*	5.4 ± 0.1	50.6 ± 1.1	44.0 ± 1.8
KTΔ*mscL*-*oprF*	6.1 ± 0.3	43.7 ± 0.8	50.2 ± 1.9
KTΔ*mscL-oprE*	5.7 ± 0.4	46.9 ± 1.5	47.4 ± 2.1
KTΔ*mscL-oprFE*	3.8 ± 0.2	48.7 ± 1.3	47.5 ± 0.9

*Values represent the mean and ±SD calculated from three independent replicates. C6, 3-hydroxyhexanoate; C8, 3-hydroxyocyanoate; C10, 3-hydroxydecanoate.*

Finally, to gain insights into the applicability of the procedure to recover proper amounts of PHA, the final cultures were resuspended (after hypotonic treatment) in non-harmful amounts of chloroform for the cell (5% v/v final concentration), and PHA was let to dissolve for 2 h. After complete chloroform evaporation, the recovered PHA was quantified as displayed in Figures 5C,D. Scarce PHA quantities, <5% over total PHA amounts, could be recovered from seven of the strains with the only exception of the KTΔ*mscL*-*oprFE* (Figures 5C,D). PHA recovery yielded 93.3% contrasted to the whole produced amounts in this strain (Figure 5D).

### Examination of Cell Wall Damage by Transmission Electron Microscopy

**FIGURE 6:**
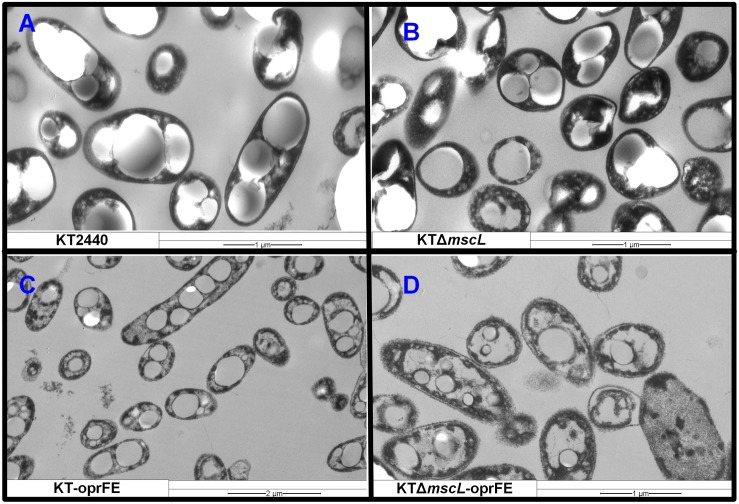
Transmission electron microscopy pictures of *Pseudomonas putida* cells producing PHA on decanoate after osmotic treatments. **(A)** KT2440, **(B)** KTΔ*mscL*, **(C)** KT-oprFE, and **(D)** KTΔ*mscL*-*oprFE* strains.

To obtain visual evidence of the provoked membrane damage, osmotic shock treatment was carried out as previously specified on the KTΔ*mscL* and wild-type strains and each bacterium carrying the *oprFE* overexpression system grown on decanoate and analyzed the samples employing transmission electron microscopy (Figure 6). The KT2440 parental and KTΔ*mscL* strains displayed the distinctive PHA granules enclosed in the cytoplasmic space, and additionally, no apparent cell wall breakage was observed in the overall bacterial population (Figures 6A,B). Overproduction of the OprFE enzymes in KT2440 did not trigger considerable wall disruption, but there is a slight alteration on the membrane (Figure 6C). Conversely, the KTΔ*mscL*-*oprFE* engineered strain clearly presented disrupted membranes in the vast majority of the cells (Figure 6D). This strain was able to produce the biopolymer similarly to the wild type, but these bacteria most likely failed to restrain it within the cellular barriers when treated with chloroform for PHA recovery (Figure 5D).

## Discussion

Here a novel system for bacterial cell lysis during biopolymer production is reported. The osmotic state of *P. putida* KT2440 in response to a combination of hypertonic and hypotonic treatments by perturbing the cell membrane was rationally engineered. First, the deletion of the *mscL* in KT2440 did not impair growth rate or total biomass yield on decanoate. Moreover, the introduction in *trans* of individual porin genes under the regulation of an inducible promoter had no restraint bacterial growth to a great extent. Both porins belong to the conserved OmpA family of proteins that constitute the major responsible players for cell turgor maintenance (Chevalier et al., 2017) and acting as non-specific transporters of ionic species (Nestorovich et al., 2006). These data confirm that the imposed challenge after salt treatment (10 g/L) did not impair significantly vital metabolic functions of the biocatalysts as recorded via cell viability (CFU) and visualization of the membrane integrity via electron microscopy (Figure 6). Most likely, this relies on the high capacity of *P. putida* to adapt itself and cope with the extracellular ever-changing osmotic state by modulating the expression of alternative valves and porins (Hancock and Brinkman, 2002). In the genome of KT2440 there are at least other eight genes encoding for MscLs (PP_4533, PP_1353, PP_5067, PP_3360, PP_0707, PP_1728, PP_5256, and PP_2741) and a wide range of porin proteins (OprE, OprC, OprJ, OprI, OprG, OprH) (Nelson et al., 2002) that could regulate the osmotic response and counteract stress. This array of osmotic stress modulators might endow *P. putida* engineered strains with a toolbox to overcome high-salt osmotic challenges, and therefore, a downshift to hypotonic environments was required. Targeted manipulation of further osmotic stress modulators might be crucial to efficiently disrupt the cell membranes of *P. putida* without the need to drastically shifting its osmotic state and might also trigger to some extent biomass loss or premature decay of cell wall integrity.

By expressing both outer membrane porins in tandem, the strains KT-*oprFE* and KTΔ*mscL*-*oprFE* displayed a slight reduction in biomass synthesis (10%) and viability after salt treatments, pointing a higher sensitivity for those strains to hypertonic shock. Many studies have reported a decrease in biomass formation when overexpressing foreign and native enzymes in the cell due to multiple factors including high energy expenditure (Borkowski et al., 2018; Shah et al., 2013) and rearrangement of the assemble machinery for enzyme synthesis (Bolognesi and Lehner, 2018). It was also striking to record a boosted biopolymer accumulation relative to the cell dry mass of the engineered strain overproducing the OprFE enzymes (Figure 5). *P. putida* and *E. coli* bacteria mediate the transport of fatty acids into the cell utilizing the porins OprF and OmpF, respectively (Tan et al., 2017). A multi-omics study reported a higher abundance of the porin OprF and OprE enzymes in *P. putida* cells when the cells produced elevated quantities of PHA (80 wt%) on decanoate in chemostat cultures (Poblete-Castro et al., 2012), which could explain to some extent the observed phenomena.

As environmental factors modulate the abundance of porins (OprF, OprC, OprD, OprH, and OprE) in bacteria of the genus *Pseudomonas* (Yamano et al., 1993), the higher production levels of these porin family proteins might turn the cell membrane more sensitive to osmotic shocks. For instance, anaerobiosis induces the expression of the *oprC* and *oprE* gene expression in *P. aeruginosa*, yet they showed no alteration to osmotic upshift (Yamano et al., 1993). As *P. putida* KT2440 is strictly aerobic, activation of the OprE in conjunction with OprF might concert a destabilization effect on the bacterial membrane. During the hypotonic shock to the salt-treated cells, a rapid loss of cell viability was only recorded in the KTΔ*mscL*-*oprFE* strain indicating that overexpression of both porins is effective in promoting cell death by abolishing the proper functioning of the MscL channel. More importantly, a biopolymer recovery of 94.2% of the biosynthesized PHA was attained. These results are consistent with our rational approach, proving that the inactivation of one of the main MscLs to relieve excessive turgor via solute secretion (Martinac et al., 1987) combined with the overproduction of the two key porin proteins (OprF and OprE) no longer sustained the physical integrity of the membrane of KT2440.

The cell lytic system developed in this study presents advantages over other self-disrupting genetic circuits implemented in the natural PHA producer *P. putida* strain. The phage holin–endolysin system has been the first strategy for biopolymer recovery with various outcomes (Hori et al., 2002; Martinez et al., 2011; Tamekou Lacmata et al., 2017). Implementation of this system in *P. putida* KT2440 have been reported to result in 16% PHA recovery of the produced biopolymer and 86% of disrupted cells, taking >15 h to complete this process (Martinez et al., 2011). Additionally, a lysozyme-based programmable lytic system had been previously devised in our group, which was very efficient in both cell disruption and PHA recuperation – 75% of the synthesized PHA – but also took >15 h to fully lysate the cell population (Borrero-De Acuna et al., 2017). The current system surpasses the available autolytic genetic systems since the cell counts diminished immediately upon hypotonic treatment leading to the complete release of the biopolymer after 3 h. This is of great importance in terms of the length of the biopolymer production process and the associated costs. Another key aspect is the similar product yields and monomer composition of the synthesized PHA between the engineered *P. putida* strain and the wild-type KT2440.

## Conclusion

In summary, a novel genetic tool to deliver controllable cell disruption upon command suitable for PHA downstream processing was developed. Importantly, PHA titers that did not differ from the biopolymer synthesized by the KT2440 wild-type strain were attained. High rates of cell lysis under PHA-producing conditions and improved biopolymer recovery in comparison to previously reported systems were demonstrated. Follow up research endeavors should target the concurrent genetic manipulation of further outer or inner membrane channels, responding to either hyper- or hypo-osmosis, to achieve cell breakage after passage through only one osmotic state. By these means, the time for cell disruption might reduce as well as the process expenses. Scaling-up the current system to render cell lyses in industrial fed-batch processes may also pose a challenge due to the high cell densities reached in bioreactors.

## Materials and Methods

### Bacterial Strain Generation

*Pseudomonas putida* KT2440 strain was employed as a reference/control strain and as background organism for genetic engineering of the designed system. All generated plasmids, primers, and strains employed throughout this work are shown in Table 1. A previously reported knockout strategy (Borrero-De Acuña et al., 2019) was used to eliminate the *mscL* gene of the KT2440 genome. The upstream flank of the *mscL* gene was amplified using the primers *mscl*UPFw and *mscl*UPRv, whereas the downstream arm was generated with the oligos *mscl*DWFw and *mscl*DWRv. Both flanking regions of the *mscL* ORF were fused by sewing PCR employing as DNA templates equal amounts of both PCR products and the oligos *mscl*UPFw and *mscl*DWRv. The spliced *mscL* upstream–downstream fragment was firstly introduced in a subcloning step into the toxin-encoding blunt end pJET1.2 commercial vector. Secondly, the *Eco*RI and *Bam*HI restriction sites present in the primer overhangs were profited for digestion and further insertion into the suicide vector pSEVA212 giving rise to pSEVA212-*mscl*UPDW. DH5αλpir cell was transformed with the constructed plasmid and kanamycin-resistant clones were browsed for proper plasmid introduction. Triparental mating was conducted using the *Escherichia coli* HB101 strain carrying the plasmid pRK600 to transfer the plasmid into KT2440 as previously specified (Benedetti et al., 2016). Kanamycin-tolerant KT2440 transconjugants were screened for genomic integration of the pSEVA212-*mscl*UPDW plasmid by PCR-amplification of the beforehand non-existing upstream–downstream spliced segment using the primers *mscl*UPFw and *mscl*DWRv. Subsequently, the I-SceI restriction enzyme-expressing plasmid pSEVA628 was introduced into the verified transconjugant and clones were selected on gentamicin plates. Expression of the I-SceI endonuclease was induced overnight by adding 15 mM m-toluic acid (Sigma-Aldrich, Darmstadt, Germany). A few resulting kanamycin-sensitive clones were PCR-assessed for proper deletion of the *mscL* gene using the combination of primers *mscl*UPFw/*mscl*DWRv and the gene aligning oligos *mscl*KOFw/*mscl*KORv. The raised strain was named KTΔ*mscL.* In parallel, the genes encoding for *oprF* and *oprE* porins were amplified from the genome of KT2440 by single-colony PCR with the oligo pairs *oprF*Fw/*oprF*Rv and *oprE*Fw/*oprE*Rv, respectively. Once again, the pJET1.2 vector was employed for subcloning purposes and both ORF were further *Eco*RI–*Xba*I (for *oprF*) and *Xba*I–*Hin*dIII (for *oprE*) inserted into the IPTG-inducible plasmid pSEVA634, which harbors a gentamicin cassette. The resulting constructs, namely pSEVA634-*oprF* and pSEVA634-*oprE*, were introduced into KT2440 and KTΔ*mscL* by triparental mating originating the following strains: KT*-oprF*, KT*-oprE*, KTΔ*mscL-oprF*, and KTΔ*mscL-oprE*. Lastly the *oprE* gene was further cleaved off the pSEVA634 plasmid with *Xba*I and *Hin*dIII and packed downstream the *oprF* gene into pSEVA634 for tandem expression of both porins. This plasmid was also incorporated into the wild-type strain and KT2440Δ*mscL* by triparental mating leading to the generation of KT-*oprFE* and KTΔ*mscL*-*oprFE*.

### Growth Conditions

*Escherichia coli* strains were incubated on Lysogeny Broth (LB) medium for sub- and cloning purposes as was *P. putida* for genetic engineering and strain construction at 37 and 30°C, respectively. The first preinoculum to carry out the bacterial PHA production started by picking one colony of the engineered and wild-type *P. putida* strains, which was suspended in 10 mL of M9 minimal medium (per liter): 12.8 g Na_2_HPO_4_ ⋅ 7H_2_O, 3 g KH_2_PO_4_, 1 g NH_4_Cl, and 0.5 g NaCl. This mixture was sterilized and supplemented with 0.12 g of MgSO_4_⋅ 7H_2_O, trace elements (6.0 FeSO_4_ ⋅ 7H_2_O, 2.7 CaCO_3_, 2.0 ZnSO_4_⋅ H_2_O, 1.16 MnSO_4_⋅ H_2_O, 0.37 CoSO_4_⋅ 7H_2_O, 0.33 CuSO_4_⋅ 5H_2_O, 0.08 H_3_BO_3_) (mg/L) (filter-sterilized), and 5 mM of decanoate as the sole carbon source. The OD_600_ values of the overnight cultures were recorded and *P. putida* cells were added to obtain an initial optical density of 0.1 in the shaking flasks. Each 500 mL Erlenmeyer flask contained 100 mL of M9 salt medium, antibiotics, trace elements, and magnesium sulfate supplemented with 20 mM of decanoate. The bacterial strains were grown in a rotary shaker set at 30°C and spinning at 180 rpm (Ecotron, INFORS HT, Switzerland). To induce porin expression IPTG (final concentration 1 mM) was added to the growing cultures at 30 h after starting the batch culture. Antibiotics were supplemented when demanded at the following final concentrations for *E. coli* (μg/mL): streptomycin 60, ampicillin 150, kanamycin 50, and gentamycin 15. For *P. putida* strains 50 mg/mL kanamycin and 50 μg/mL gentamicin were added.

### Cell Surface Hydrophobicity Assay

The bacterial adhesion to hydrocarbon assay (BAHA) was performed as described by Reifsteck et al. (1987) with slight modifications. Briefly, after 48 h of starting the batch, 2 mL of cultures was centrifuged at 4,000 × *g* for 10 min at 4°C, the supernatant discarded, and the cells resuspended in the same volume with phosphate buffered saline (PBS). This step was repeated two more times, and finally resuspended in PBS to reach an OD_600_ of 0.4. To a 2.5 mL of cell suspension in a glass tube, 0.5 mL of *p*-xylene was added and vigorously stirred using a vortex for 1 min, and finally held for 30 min at room temperature to allow hydrocarbon separation. The absorbance (OD_600_) of the aqueous phase was measured before (*A*_0_) and after the treatment (*A*), and the BAHA calculated using the following formula (%): 100.[1−(*A*/*A*_0_)].

### Outer Membrane Permeability Assay

After culture for 48 h, 2 mL of the *P. putida* cells was washed three times with PBS buffer and resuspended with the same buffer to reach an optical density of (OD_600_) of 0.2. Subsequently, 10 μL of *N*-phenyl-1-naphthylamine (NPN) stock solution 1 mM in 1 M Tris-HCl was mixed with 1 mL of the cell suspension. The mixture reacted for 2 min at room temperature and 0.1 mL of 1 M KH_2_PO_4_ was added to terminate the reaction. The florescence intensity (excitation at 355 nm) was recorded by inspecting the emission spectrum from 390 to 490 nm using a multiplate reader (Synergy H1, Biotek, United States).

### Colony Forming Unit Counting

*Pseudomonas putida* KT2440, and recombinant strains were incubated in M9 medium supplemented with 20 mM decanoate in shaking flasks. After 30 h of growth, expression of the corresponding porins was induced by adding 1 mM IPTG. Gene over-expression was permitted for 18 h. Prior to salt treatment cell viability was monitored by conducting serial dilutions of each culture in PBS and plating 100 μL on LB with 2.5% w/v agar. CFU were counted on each dilution. Immediately after, 10 g/L of sterile NaCl was added to the growing cultures. The hyper-osmotic shock was allowed for 1 h and CFU counted as above specified. Subsequently, the entire cell culture was centrifuged at room temperature (5,000 × *g* for 5 min), the supernatant discarded, and cells resuspended in 100 mL of distilled water. Afterward, the CFU again numbered over 3 h period.

### FACS Analysis

Flow cytometry analysis performed on a FACS ARIA FUSION cell sorter (BD Biosciences, San Jose, CA, United States) equipped with two lasers with excitation wavelengths of 488 and 633 nm. An instrument quality control was carried out by using FACS DIVA CS&T Research beads (BD Biosciences, San Jose, CA, United States) prior to bacteria introduction, following the manufacturer’s instructions. Preliminary tests were conducted on the flow cytometer to rule out detection of cells debris or other components that might be present on the media culture or FACS Staining buffer (PBS, Tween 20 at 0.01%). Thereby, the noise effect, voltage, and threshold settings were determined. According to the provider’s guidelines bacterial cultures were stained with BD Cell Viability Kit (CAT #349483 BD Biosciences, San Jose, CA, United States). In brief, approximately 5 × 10^6^ bacteria/mL were transferred to a 12 × 75 mm polystyrene test tube and resuspended in 200 μL of FACS staining buffer. Two sorts of control samples were included in the assessment: non-lysed cells or bacterial culture lysates undergoing to heat-cold shock treatment (five cycles of freezing at −80°C for 3 min followed by 3 min of incubation at 90°C). By these means the instruments settings and dead cells gate/threshold were established. A staining mixture was set up by reverse pipetting by adding 5 μL of Thiazole Orange solution 17 μM and 5 μL of propidium iodide solution 19 mM. The samples were lastly acquired on the FACS Aria Fusion instrument after 5 min incubation time.

### PHA Quantification and Characterization

Methanolysis of lyophilized cell dry mass (5–10 mg) was introduced in sealed tubes containing 2 mL chloroform, 2 mL methanol, 15% (v/v) H_2_SO_4_, and 0.5 (mg/mL) 3-methylbenzoic acid. The tubes were incubated for 4 h at 100°C in a thermoblock. After the tubes reached room temperature, 1 mL of miliQ water was mixed with the reaction solution, and vigorously agitated for 1 min. The mixture was then transferred to a Falcon tube (15 mL) and centrifuged for 10 min at 6,000 × *g*. The lower part of the biphasic solution, containing the methyl esters of the biopolymer, was separated and analyzed via gas chromatography coupled to mass spectrometry (YL6900, Young Instruments, South Korea) using the methodology previously described by Borrero-De Acuña et al. (2019). Briefly, a calibration curve was created using a purified medium-chain-length-PHA synthesized in a previous work (Oliva-Arancibia et al., 2017) to interpolate data samples. Once the retention time of the peaks was contrasted with the standards, their chemical structures were characterized based on the resulting mass compatibility (NIST 17 Mass Spectral library). The percentage of PHA in the cell was defined as the amount of the biopolymer divided by the total cell dry mass and multiplied by 100.

### Biopolymer Recovery

In order to determine the PHA recovery of each *P. putida* strain, 36 mL culture was blended with 2 mL of high-grade chloroform at a final concentration of 5% (v/v). Subsequently, the mixture was stirred at room temperature for 2 h and further centrifuged at 3,400 × *g* for 10 min at 4°C. Thereby, PHA precipitation was avoided and a phase constituted of chloroform-PHA was generated. The aforesaid phase was retrieved with a Pasteur pipette and maintained at room temperature until the chloroform evaporated. The recovered PHA was transferred to 500 μL chloroform and quantified as described in the prior section.

### Electron Microscopy Operation

Fixation of chilled cultures was driven by addition of glutaraldehyde and formaldehyde at 2 and 5%, respectively. Washing was carried out with cacodylate buffer (0.01 mol/L) 1 cacodylate (0.01 mol/L), 1 CaCl_2_ (0.01 mol/L), 1 MgCl_2_ 6H_2_O (0.09 mol/L), 1 sucrose, pH 6/9) being the samples stained with aqueous osmium for 1 h at room temperature. Samples were subsequently dried out gradually with acetone at the increasing concentrations of 10, 30, 50, 70, 90, and 100%. Each dehydration step grade with acetone was performed for 30 min with the exception of the 70% acetone step that was conducted overnight mixed with 2% uranyl acetate. Exposy was the resin of choice being infiltrated following the Spurr method for hard resin for several days. Cells were sliced into 40 nm ultrathin sections with a diamond knife and further counterstained with uranyl acetate and lead citrate. The resulting samples were analyzed operating a TEM910 transmission electron microscope (Carl Zeiss, Oberkochen, Germany) at an acceleration voltage of 80 kV. Digital images were taken with Slow-Scan CCD-Camera (ProScan, 1024 × 1024, Scheuring, Germany) with ITEM-Software (Olympus Soft Imaging Solutions, Munster, Germany) and recorded onto a MO-disc. Adobe Photoshop was used to fine-tune contrast and brightness.

## Data Availability Statement

All datasets generated for this study are included in the article/supplementary material.

## Author Contributions

MO-S, AC, CA-C, and NP conducted the experimental work. JB-DA and IP-C designed the experiments, analyzed the data, and wrote the manuscript.

## Conflict of Interest

The authors declare that the research was conducted in the absence of any commercial or financial relationships that could be construed as a potential conflict of interest.
